# Proteomic distinction of renal oncocytomas and chromophobe renal cell carcinomas

**DOI:** 10.1186/s12014-018-9200-6

**Published:** 2018-08-03

**Authors:** Vanessa Drendel, Bianca Heckelmann, Christoph Schell, Lucas Kook, Martin L. Biniossek, Martin Werner, Cordula A. Jilg, Oliver Schilling

**Affiliations:** 1 Institute for Surgical Pathology, Medical Center – University of Freiburg, Faculty of Medicine, University of Freiburg, Freiburg, Germany; 2 Institute of Molecular Medicine and Cell Research, Faculty of Medicine, University of Freiburg, Stefan Meier Strasse 17, 79104 Freiburg, Germany; 3 German Cancer Consortium (DKTK) and German Cancer Research Center (DKFZ), Heidelberg, Germany; 4 Department of Urology, Medical Center – University of Freiburg, Faculty of Medicine, University of Freiburg, Hugstetter Strasse 55, 79106 Freiburg, Germany; 5 Comprehensive Cancer Center Freiburg, Medical Center – University of Freiburg, Freiburg, Germany; 6 BIOSS Centre for Biological Signaling Studies, University of Freiburg, 79104 Freiburg, Germany

**Keywords:** Renal cell tumors, Formalin-fixation, Paraffin embedment, Proteomics, Immunohistochemistry

## Abstract

**Background:**

Renal oncocytomas (ROs) are benign epithelial tumors of the kidney whereas chromophobe renal cell carcinoma (chRCCs) are malignant renal tumors. The latter constitute 5–7% of renal neoplasias. ROs and chRCCs show pronounced molecular and histological similarities, which renders their differentiation demanding. We aimed for the differential proteome profiling of ROs and early-stage chRCCs in order to better understand distinguishing protein patterns.

**Methods:**

We employed formalin-fixed, paraffin-embedded samples (six RO cases, six chRCC cases) together with isotopic triplex dimethylation and a pooled reference standard to enable cohort-wide quantitative comparison. For lysosomal-associated membrane protein 1 (LAMP1) and integrin alpha-V (ITGAV) we performed corroborative immunohistochemistry (IHC) in an extended cohort of 42 RO cases and 31 chRCC cases.

**Results:**

At 1% false discovery rate, we identified > 3900 proteins, of which > 2400 proteins were consistently quantified in at least four RO and four chRCC cases. The proteomic expression profiling discriminated ROs and chRCCs and highlighted established features such as accumulation of mitochondrial proteins in ROs together with emphasizing the accumulation of endo-lysosomal proteins in chRCCs. In line with the proteomic data, IHC showed enrichment of LAMP1 in chRCC and of ITGAV in RO.

**Conclusion:**

We present one of the first differential proteome profiling studies on ROs and chRCCs and highlight differential abundance of LAMP1 and ITGAV in these renal tumors.

**Electronic supplementary material:**

The online version of this article (10.1186/s12014-018-9200-6) contains supplementary material, which is available to authorized users.

## Background

Chromophobe renal cell carcinoma (chRCC) constitute 5–7% of renal neoplasias [1]. ChRCC are thought to originate from cell of the distal nephron [2]. Their overall prognosis is more favourable than for renal clear cell carcinomas with a 5-year survival rate of > 75% [1, 3]. However, this is still a malignant tumor entity with the potential for recurrence or metastatic spread. Renal oncocytomas (ROs) are benign epithelial tumors of the kidney. They constitute up to 7% of all adult renal tumors [4]. ROs have been first described as late as 1942 [5] and clinical reports have remained scarce until the 1970s [6]. Differentiation between RO and chRCC in pathological routine practice is often considered challenging [4, 7]. This is because of strong similarities in morphology, growth pattern and localization of benign ROs and malignant chRCCs. For this reason, distinguishing biomarkers are actively being researched. In this context, system-wide, omics-type expression profiling studies are emerging as a strong and unbiased approach. Several of such expression studies aimed at a comprehensive differential profiling of various renal cell tumors, also including clear cell renal cell carcinoma (ccRCCs) or papillary renal cell carcinoma. Often, such studies involved only low numbers of RO and chRCC cases and aimed at their collective distinction from other renal cell tumors rather than molecularly distinguishing ROs and chRCCs.

Yusenko et al. probed genome alterations and DNA copy number variants to specifically differentiate ROs and chRCCs. They identified several genomic alterations that differ between chRCCs and ROs [8]. A second genomic study identified differing chromosomal abnormalities with regard to chromosome 19 in ROs or chRCCs [9]. The resulting gene expression effects affected oxygen sensing. This is an intriguing parallel to ccRCCs which frequently carry somatic, inactivating mutations of the *Von Hippel*-*Lindau* gene, ultimately leading to the expression of hypoxia-related genes and promotion of tumorigenesis [10]. On the genomic level, Joshi et al. [11] distinguish two types of ROs and link chromosomal abnormalities involving chromosome 1, X or Y, and/or 14 and 21 with the potential for progression from RO to chRCC. Rohan et al. [12] performed a global transcriptomic profiling of ROs and chRCCs. Major expression differences were found with regard to transcript encoding proteins involved in vesicular transport and cell junction. On the protein level, multiple protein biomarkers have been suggested (reviewed in [4]) but to date there has not been an unbiased, differential proteomic profiling of ROs and chRCCs. However, proteomic profiling is gaining interest for the investigation of malignancies due to the limited correlation between mRNA and protein levels [13, 14]. Formalin-fixed, paraffin-embedded (FFPE) samples are a valuable resource for proteomic profiling [15–17], enabling retrospective profiling of clinic-pathologically annotated specimens [18, 19]. In the present study, we employed “FFPE proteomics” for the differential proteomic profiling of RO and chRCC cases for which we find noticeable differences. In a larger cohort comprising > 70 RO and chRCC cases, we corroborate elevated presence of lysosomal-associated membrane protein 1 in chRCCs and elevated presence of integrin alpha-V in ROs.

## Methods

### Ethics statement

The study was approved by the Ethics Committee of the University Medical Center Freiburg (311/12). Before study inclusion, all patient data were anonymized.

### Histopathological diagnosis for renal oncocytoma or chromophobe renal cell carcinoma

The diagnosis for all RO or chRCC cases used in this study was based on histopathologic parameters (cytoplasm, cell membrane, perinuclear halo, tumor border and septae) and corroborated by immunohistochemistry (IHC) for CD117, cytokeratin-7, and vimentin. These IHC stainings were part of the routine immunohistopathological diagnosis with the corresponding antibodies being supplied by Dako (Hamburg, Germany). We focused on chRCC cases that displayed diffuse-membranous expression of cytokeratin-7 whereas its expression was largely absent in the RO cases [1, 7]. Moreover, the RO and chRCC cases displayed membranous expression of CD117 [7]. Finally, the RO and chRCC cases were vimentin-negative [1].

### Tissue collection, sample preparation, liquid chromatography-tandem mass spectrometry (LC–MS/MS), and data analysis

FFPE tissue specimens of six ROs and six chRCCs were used as described previously [15, 19], including microscopically controlled macrodissection to remove areas of necrosis, fibrosis, hemorrhage, and inflammation. For quantitative comparison, triplex isotopic dimethylation of primary amines was employed [20], distinguishing RO, chRCC, and a pooled mix that serves as a standard similar to the Super-SILAC approach [21]. Samples were further fractionated by strong cation exchange chromatography as described [22]. LC–MS/MS was performed using a Q-Exactive plus (Thermo Scientific) mass spectrometer coupled to an Easy nanoLC 1000 (Thermo Scientific) as described previously [18]. MS data were analyzed by MaxQuant version 1.5.28 [23] as described previously [19]. Proteins were only further considered if they were identified and quantified in at least four RO samples and four chRCC samples. Due to this strict requirement, we also included proteins that were identified and quantified by single peptides in individual samples. Files obtained by MaxQuant were further processed using RStudio v.0.99.446 (R Foundation for Statistical Computing, Vienna, Austria) as previously described [24]. Reverse and potential contaminants entries were removed. Ratios were log_2_ transformed, normalized by centering, and a linear model was fitted using the limma package [25].

### Immunohistochemical analysis

IHC analysis was performed with an extended patient cohort, comprising 42 RO cases and 31 chRCC cases. IHC was performed for lysosomal-associated membrane protein 1 (LAMP1) and integrin alpha-V (ITGAV). Slices of 2 µm thickness from FFPE tissue samples were prepared using a Leica RM2255 microtome. Heat induced antigen retrieval was performed at pH 9.0. Primary antibodies were rabbit polyclonal to LAMP1 (Abcam, ab24170, stock concentration 1.0 mg/ml) or rabbit monoclonal to ITGAV (Abcam, EPR16800, stock concentration 1.0 mg/ml). For incubation, primary antibodies were diluted in Zytomed dilution buffer (ZUC025-500; 1:300 for the LAMP1 antibody, 1:7000 for the ITGAV antibody). Incubation time was 60 min. Visualization was performed using DAKO Envision Flex+, Mouse, high pH (Link) Detecting System (K800221-2). Sections were counterstained with hematoxylin for 1 min, dehydrated in an ascending alcohol concentration and covered with xylol and coverslipping film (Tissue-Tek^R^ 4770). For evaluation, two experienced pathologists reviewed LAMP1 and ITGAV expression in RO or chRCC tumor cells using a semi-quantitative scoring system covering absence of signal (score 0), weak detection (score 1), medium detection (score 2), and strong detection (score 3). For every sample, the fractional area of the different detection levels (scores 0–3) was determined and reported as percentage of the total tumor area under investigation.

## Results and discussion

### Experimental set-up

**Fig. 1:**
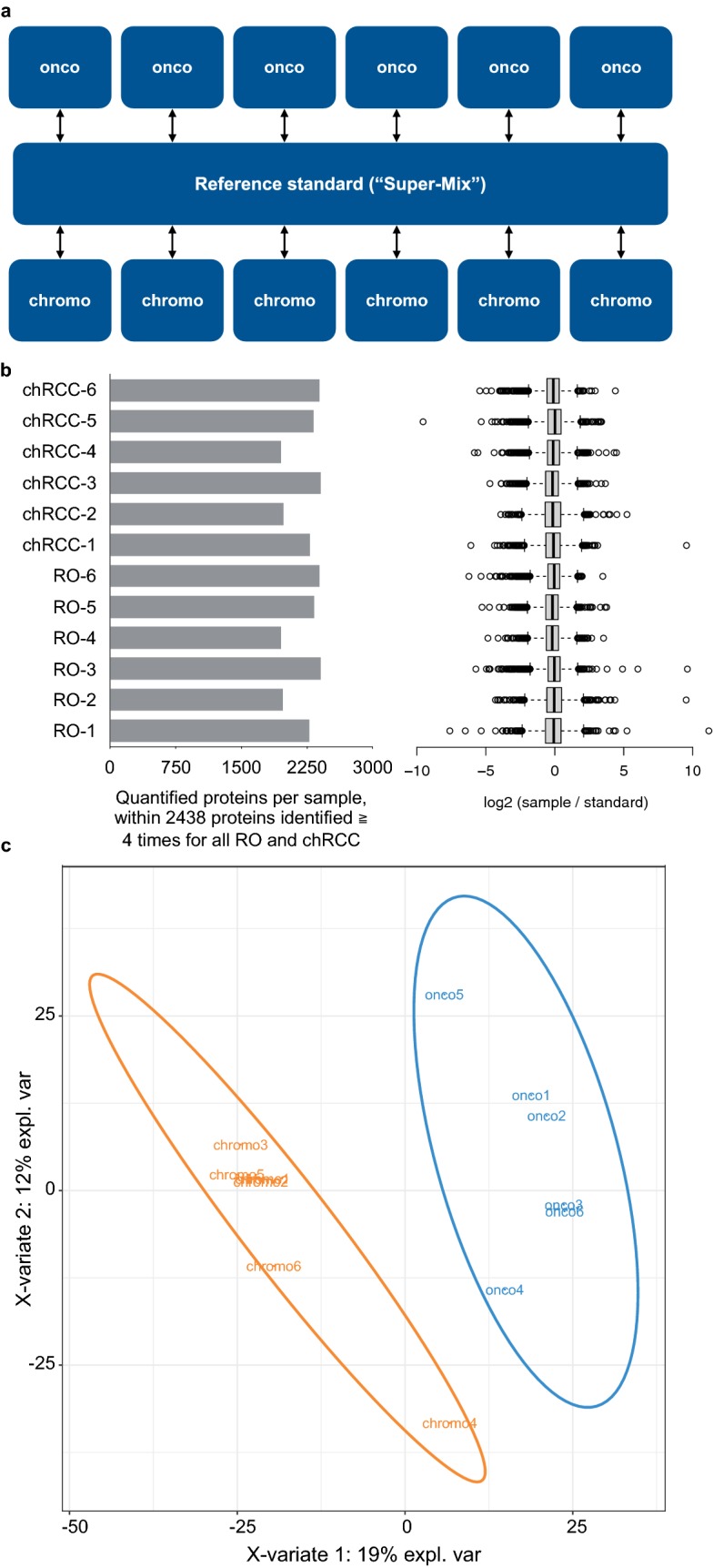
**a** Samples of chRCC tissue or RO tissue were collected from FFPE specimens and, post-trypsination, differentially labeled by isotopic, formaldehyde-based dimethylation. A differentially labeled, pooled reference standard was also included. **b** > 2400 proteins were identified (false-discovery rate < 1%) and quantified in at least four RO and four chRCC samples. Within this core proteome, each chRCC and RO case contributed a comparable number of protein identifications and quantitations. Also, the log-transformed sample/standard ratios were comparable for all cases. Whiskers extend to data points that are less than 1.5 × IQR away from 1st/3rd quartile. **c** Supervised partial least squares discriminant analysis separates the RO and chRCC proteome profiles

We aimed for a differential, quantitative proteome characterization of RO and chRCC using FFPE samples for which we have shown amenability to quantitative proteomic analysis using isotope-coded dimethylation [15]. Our cohort for the proteomic analysis comprised 12 cases; six RO and six chRCC. In order to enable cohort-wide comparison, we also included a pooled sample (comprised of RO and chRCC tissue) as a spike-in reference standard against which every other sample can be compared. This set-up is reminiscent of the Super-SILAC technique, in which metabolically labelled proteomes serve as spike-in reference standard [21]. A triplex labeling scheme was employed (Fig. 1a, actual labeling setup in Additional file 1: Table S1). In a second cohort (42 RO cases; 31 chRCC cases) we employed immunohistochemistry to probe the expression of LAMP1 and ITGAV. Since the benign oncocytomas are very rarely invasive (which constitutes their benign nature), we focused on chRCC cases for which tumor growth was still confined to the kidney (T1 or T2). An overview of the patient characteristics is provided in Table 1.

**Table 1:** Patient characteristics

	Proteomics		Immunohistochemistry (*no* overlap with cases used for proteomic analysis)	
	Renal oncocytomas	Chromophobe renal cell carcinomas	Renal oncocytomas	Chromophobe renal cell carcinomas
N	6	6	42	31
Tumor size/T classification				
≤ 4 cm/1a	5	4	25	21
≤ 7 cm/1b	1	2	15	6
≤ 10 cm/2a	0	0	1	3
> 10 cm/2b	0	0	1	1
Number of simultaneous tumors^a^				
1	6	6	38	31
2	0	0	3	0
6	0	0	1	0
Affected kidney				
Left	1	2	21	16
Right	4	3	19	12
Not documented	1	1	2	3
Gender				
Male	3	3	24	15
Female	3	3	18	16
Age				
Average	66	56	64	60
Range	57–80	42–74	42–88	34–83

^a^If simulatenous tumors were present, only one tumor per patient was used for the analyses

### Proteome profiling of RO and chRCC

LC–MS/MS analysis enabled the identification (at a false discovery rate < 1%) of > 3900 proteins. Of these, > 2400 proteins were identified and quantified in at least four RO and four chRCC cases. Within this “core proteome”, each case allowed for the identification and quantification of a comparable number of proteins (Fig. 1b). Likewise, the protein quantitation profiles (log-transformed sample/standard ratios) are similar (Fig. 1b). We conclude that proteome coverage and—quantitation allows for the comparison of the RO and chRCC proteome profiles. We employed partial least squares discriminant analysis (PLS-DA) as an initial step to probe whether RO and chRCC display distinguishable proteome profiles. Supervised PLS-DA of the proteomic dataset (Fig. 1c) clearly separates RO and chRCC in an unbiased manner, thus corroborating their initial distinction. Noteworthy, RO and chRCC were primarily distinguished by histopathological parameters and cytokeratin-7 IHC as part of routine diagnosis. Further approaches such as S100A1 IHC or Hale colloidal iron staining [7] have not been routinely employed, which however is not meant to question their discriminatory power.

### Quantitative proteome differences and distinct proteome motifs of RO and chRCC

**Fig. 2:**
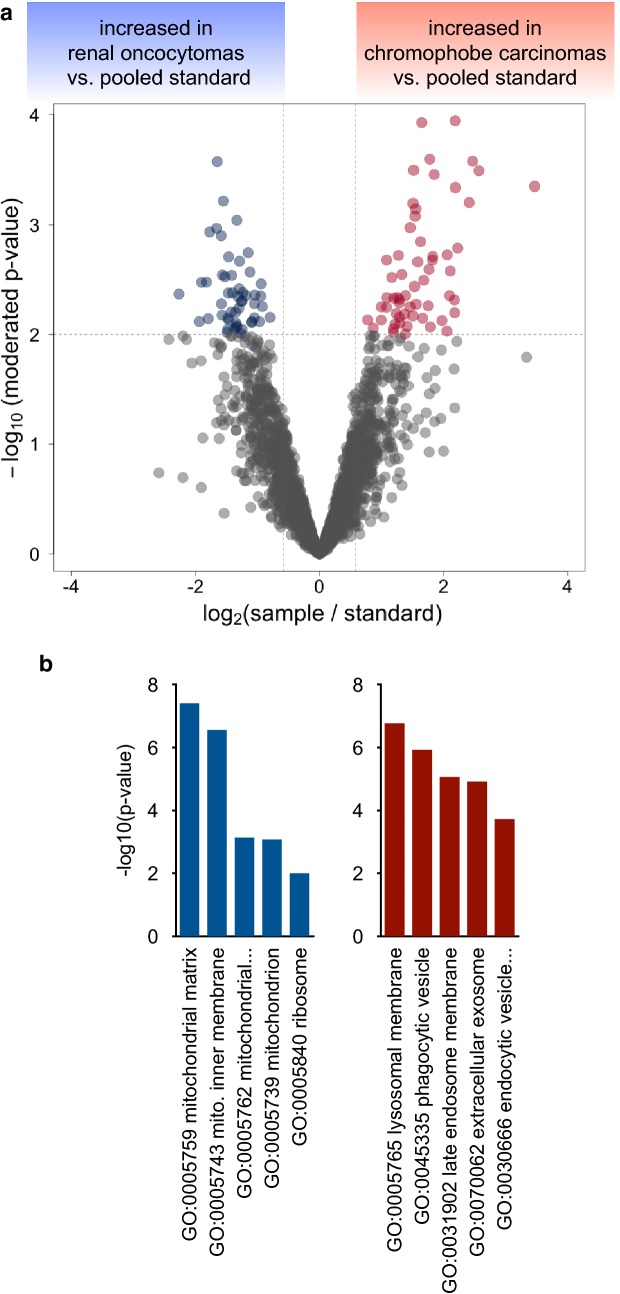
**a** Statistical analysis using linear models as implemented in Limma distinguishes significantly (p < 0.01) affected proteins. **b** Gene ontology (confined to “cellular compartment”) analysis of proteins that were found to be significantly increased in either RO or chRCC

To identify proteins that are significantly enriched in either RO or chRCC (as compared to the pooled standard), we employed a linear model as implemented in the limma statistical package [26], which is particularly powerful with regard to multiple testing correction and prevention of false-positive discoveries in the analysis of omics-style data. We consider proteins with a limma moderated *p* value < 0.01 and protein identification based on at least three peptide-spectrum-matches to be significantly enriched in either RO or chRCC (as compared to the pooled standard). These criteria resulted in 51 proteins being significantly enriched in RO tissue and 59 proteins being significantly enriched in chRCC tissue (Tables 2 and 3). The corresponding volcano plot is visualized in Fig. 2a; all limma moderated p-values and average log2 ratios are listed in Additional file 2: Table S2. In line with earlier reports [1, 7] and our IHC-assisted confirmation of RO or chRCC diagnosis, there was significant enrichment of cytokeratin-7 in the chRCC cases (p ≪ 0.01).

**Table 2:** Proteins found to be significantly enriched in RO

Average log2 (RO/standard)	Average log2 (chRCC/standard)	p-value (limma moderated)	Uniprot ID	Protein names
1.09	− 0.57	0.00	P06756	Integrin alpha-V (Vitronectin receptor) (Vitronectin receptor subunit alpha) (CD antigen CD51) [Cleaved into: Integrin alpha-V heavy chain; Integrin alpha-V light chain]
0.35	− 1.21	0.00	Q9H9J2	39S ribosomal protein L44, mitochondrial (L44mt) (MRP-L44) (EC 3.1.26.-) (Mitochondrial large ribosomal subunit protein mL44)
0.46	− 0.87	0.00	O15382	Branched-chain-amino-acid aminotransferase, mitochondrial (BCAT(m)) (EC 2.6.1.42) (Placental protein 18) (PP18)
0.38	− 1.28	0.00	P51398	28S ribosomal protein S29, mitochondrial (MRP-S29) (S29mt) (Death-associated protein 3) (DAP-3) (Ionizing radiation resistance conferring protein) (Mitochondrial small ribosomal subunit protein mS29)
0.40	− 1.38	0.00	P16219	Short-chain specific acyl-CoA dehydrogenase, mitochondrial (SCAD) (EC 1.3.8.1) (Butyryl-CoA dehydrogenase)
0.42	− 1.17	0.00	Q6PI48	Aspartate–tRNA ligase, mitochondrial (EC 6.1.1.12) (Aspartyl-tRNA synthetase) (AspRS)
0.50	− 0.66	0.00	Q9BYD6	39S ribosomal protein L1, mitochondrial (L1 mt) (MRP-L1) (Mitochondrial large ribosomal subunit protein uL1m)
0.36	− 1.11	0.00	P30048	Thioredoxin-dependent peroxide reductase, mitochondrial (EC 1.11.1.15) (Antioxidant protein 1) (AOP-1) (HBC189) (Peroxiredoxin III) (Prx-III) (Peroxiredoxin-3) (Protein MER5 homolog)
0.33	− 0.96	0.00	P50897	Palmitoyl-protein thioesterase 1 (PPT-1) (EC 3.1.2.22) (Palmitoyl-protein hydrolase 1)
0.33	− 0.79	0.00	Q96GK7	Fumarylacetoacetate hydrolase domain-containing protein 2A (EC 3.-.-.-)
0.04	− 1.53	0.00	P55084	Trifunctional enzyme subunit beta, mitochondrial (TP-beta) [Includes: 3-ketoacyl-CoA thiolase (EC 2.3.1.16) (Acetyl-CoA acyltransferase) (Beta-ketothiolase)]
0.40	− 1.02	0.00	Q96EL3	39S ribosomal protein L53, mitochondrial (L53mt) (MRP-L53) (Mitochondrial large ribosomal subunit protein mL53)
0.47	− 1.06	0.00	Q96PE7	Methylmalonyl-CoA epimerase, mitochondrial (EC 5.1.99.1) (DL-methylmalonyl-CoA racemase)
0.04	− 1.79	0.00	Q8TCS8	Polyribonucleotide nucleotidyltransferase 1, mitochondrial (EC 2.7.7.8) (3′–5′ RNA exonuclease OLD35) (PNPase old-35) (Polynucleotide phosphorylase 1) (PNPase 1) (Polynucleotide phosphorylase-like protein)
0.97	− 0.93	0.00	Q96P44	Collagen alpha-1(XXI) chain
0.33	− 0.61	0.00	P08559	Pyruvate dehydrogenase E1 component subunit alpha, somatic form, mitochondrial (EC 1.2.4.1) (PDHE1-A type I)
0.47	− 0.83	0.00	Q8N5M1	ATP synthase mitochondrial F1 complex assembly factor 2 (ATP12 homolog)
0.23	− 1.01	0.00	Q8N0X4	Citramalyl-CoA lyase, mitochondrial (EC 4.1.3.25) (Beta-methylmalate synthase) (EC 2.3.3.-) (Citrate lyase subunit beta-like protein) (Citrate lyase beta-like) (Malate synthase) (EC 2.3.3.9)
0.41	− 1.00	0.00	Q9HD33	39S ribosomal protein L47, mitochondrial (L47mt) (MRP-L47) (Mitochondrial large ribosomal subunit protein uL29 m) (Nasopharyngeal carcinoma metastasis-related protein 1)
0.47	− 1.00	0.00	Q16822	Phosphoenolpyruvate carboxykinase [GTP], mitochondrial (PEPCK-M) (EC 4.1.1.32)
1.01	− 1.26	0.00	P02792	Ferritin light chain (Ferritin L subunit)
0.27	− 0.77	0.00	Q9UIJ7	GTP:AMP phosphotransferase AK3, mitochondrial (EC 2.7.4.10) (Adenylate kinase 3) (AK 3) (Adenylate kinase 3 alpha-like 1)
0.46	− 0.73	0.00	Q9NYK5	39S ribosomal protein L39, mitochondrial (L39mt) (MRP-L39) (39S ribosomal protein L5, mitochondrial) (L5mt) (MRP-L5) (Mitochondrial large ribosomal subunit protein mL39)
0.37	− 0.58	0.00	P11177	Pyruvate dehydrogenase E1 component subunit beta, mitochondrial (PDHE1-B) (EC 1.2.4.1)
0.18	− 1.13	0.00	Q9UFN0	Protein NipSnap homolog 3A (NipSnap3A) (Protein NipSnap homolog 4) (NipSnap4) (Target for Salmonella secreted protein C) (TassC)
0.36	− 0.89	0.00	P12694	2-oxoisovalerate dehydrogenase subunit alpha, mitochondrial (EC 1.2.4.4) (Branched-chain alpha-keto acid dehydrogenase E1 component alpha chain) (BCKDE1A) (BCKDH E1-alpha)
0.52	− 0.75	0.01	Q9Y3B7	39S ribosomal protein L11, mitochondrial (L11 mt) (MRP-L11) (Mitochondrial large ribosomal subunit protein uL11m)
0.33	− 0.72	0.01	Q8N490	Probable hydrolase PNKD (EC 3.-.-.-) (Myofibrillogenesis regulator 1) (MR-1) (Paroxysmal nonkinesiogenic dyskinesia protein) (Trans-activated by hepatitis C virus core protein 2)
0.54	− 1.05	0.01	P52815	39S ribosomal protein L12, mitochondrial (L12 mt) (MRP-L12) (5c5-2) (Mitochondrial large ribosomal subunit protein bL12m)
0.40	− 0.53	0.01	P26038	Moesin (Membrane-organizing extension spike protein)
0.67	− 0.64	0.01	Q9Y619	Mitochondrial ornithine transporter 1 (Solute carrier family 25 member 15)
0.22	− 1.21	0.01	P22033	Methylmalonyl-CoA mutase, mitochondrial (MCM) (EC 5.4.99.2) (Methylmalonyl-CoA isomerase)
0.60	− 0.76	0.01	P20674	Cytochrome c oxidase subunit 5A, mitochondrial (Cytochrome c oxidase polypeptide Va)
0.80	− 0.77	0.01	Q15111	Inactive phospholipase C-like protein 1 (PLC-L1) (Phospholipase C-deleted in lung carcinoma) (Phospholipase C-related but catalytically inactive protein) (PRIP)
0.40	− 0.40	0.01	P27348	14-3-3 protein theta (14-3-3 protein T-cell) (14-3-3 protein tau) (Protein HS1)
0.29	− 0.76	0.01	Q5TEU4	Arginine-hydroxylase NDUFAF5, mitochondrial (EC 1.-.-.-) (NADH dehydrogenase [ubiquinone] 1 alpha subcomplex assembly factor 5) (Putative methyltransferase NDUFAF5) (EC 2.1.1.-)
0.01	− 1.47	0.01	P10809	60 kDa heat shock protein, mitochondrial (EC 3.6.4.9) (60 kDa chaperonin) (Chaperonin 60) (CPN60) (Heat shock protein 60) (HSP-60) (Hsp60) (HuCHA60) (Mitochondrial matrix protein P1) (P60 lymphocyte protein)
0.62	− 1.18	0.01	Q96CU9	FAD-dependent oxidoreductase domain-containing protein 1 (EC 1.-.-.-)
0.46	− 1.01	0.01	P10606	Cytochrome c oxidase subunit 5B, mitochondrial (Cytochrome c oxidase polypeptide Vb)
0.33	− 0.77	0.01	P04179	Superoxide dismutase [Mn], mitochondrial (EC 1.15.1.1)
1.09	− 0.85	0.01	O14773	Tripeptidyl-peptidase 1 (TPP-1) (EC 3.4.14.9) (Cell growth-inhibiting gene 1 protein) (Lysosomal pepstatin-insensitive protease) (LPIC) (Tripeptidyl aminopeptidase) (Tripeptidyl-peptidase I) (TPP-I)
0.34	− 0.63	0.01	P21953	2-oxoisovalerate dehydrogenase subunit beta, mitochondrial (EC 1.2.4.4) (Branched-chain alpha-keto acid dehydrogenase E1 component beta chain) (BCKDE1B) (BCKDH E1-beta)
0.25	− 0.85	0.01	P03928	ATP synthase protein 8 (A6L) (F-ATPase subunit 8)
0.18	− 1.17	0.01	P30042	ES1 protein homolog, mitochondrial (Protein GT335) (Protein KNP-I)
0.44	− 0.90	0.01	P09001	39S ribosomal protein L3, mitochondrial (L3 mt) (MRP-L3) (Mitochondrial large ribosomal subunit protein uL3m)
0.80	− 0.55	0.01	Q15067	Peroxisomal acyl-coenzyme A oxidase 1 (AOX) (EC 1.3.3.6) (Palmitoyl-CoA oxidase) (Straight-chain acyl-CoA oxidase) (SCOX)
0.37	− 1.01	0.01	O75947	ATP synthase subunit d, mitochondrial (ATPase subunit d)
0.29	− 0.97	0.01	P82933	28S ribosomal protein S9, mitochondrial (MRP-S9) (S9 mt) (Mitochondrial small ribosomal subunit protein uS9m)
0.38	− 1.11	0.01	P09669	Cytochrome c oxidase subunit 6C (Cytochrome c oxidase polypeptide VIc)
0.38	− 1.11	0.01	Q3ZCW2	Galectin-related protein (Lectin galactoside-binding-like protein)
0.37	− 0.90	0.01	Q9Y3D9	28S ribosomal protein S23, mitochondrial (MRP-S23) (S23mt) (Mitochondrial small ribosomal subunit protein mS23)
1.09	− 0.57	0.00	P06756	Integrin alpha-V (Vitronectin receptor) (Vitronectin receptor subunit alpha) (CD antigen CD51) [Cleaved into: Integrin alpha-V heavy chain; Integrin alpha-V light chain]
0.35	− 1.21	0.00	Q9H9J2	39S ribosomal protein L44, mitochondrial (L44mt) (MRP-L44) (EC 3.1.26.-) (Mitochondrial large ribosomal subunit protein mL44)
0.46	− 0.87	0.00	O15382	Branched-chain-amino-acid aminotransferase, mitochondrial (BCAT(m)) (EC 2.6.1.42) (Placental protein 18) (PP18)
0.38	− 1.28	0.00	P51398	28S ribosomal protein S29, mitochondrial (MRP-S29) (S29mt) (Death-associated protein 3) (DAP-3) (Ionizing radiation resistance conferring protein) (Mitochondrial small ribosomal subunit protein mS29)
0.40	− 1.38	0.00	P16219	Short-chain specific acyl-CoA dehydrogenase, mitochondrial (SCAD) (EC 1.3.8.1) (Butyryl-CoA dehydrogenase)
0.42	− 1.17	0.00	Q6PI48	Aspartate–tRNA ligase, mitochondrial (EC 6.1.1.12) (Aspartyl-tRNA synthetase) (AspRS)
0.50	− 0.66	0.00	Q9BYD6	39S ribosomal protein L1, mitochondrial (L1 mt) (MRP-L1) (Mitochondrial large ribosomal subunit protein uL1m)

**Table 3:** Proteins found to be significantly enriched in chRCC

Average log2 (RO/standard)	Average log2 (chRCC/standard)	p-value (limma moderated)	Uniprot ID	Protein names
− 0.83	0.59	0.01	P11117	Lysosomal acid phosphatase (LAP) (EC 3.1.3.2)
− 0.66	0.21	0.01	P04075	Fructose-bisphosphate aldolase A (EC 4.1.2.13) (Lung cancer antigen NY-LU-1) (Muscle-type aldolase)
− 0.81	0.27	0.01	P09972	Fructose-bisphosphate aldolase C (EC 4.1.2.13) (Brain-type aldolase)
− 0.82	0.38	0.00	P07355	Annexin A2 (Annexin II) (Annexin-2) (Calpactin I heavy chain) (Calpactin-1 heavy chain) (Chromobindin-8) (Lipocortin II) (Placental anticoagulant protein IV) (PAP-IV) (Protein I) (p36)
− 1.06	0.52	0.00	Q96BM9	ADP-ribosylation factor-like protein 8A (ADP-ribosylation factor-like protein 10B) (Novel small G protein indispensable for equal chromosome segregation 2)
− 0.73	0.83	0.01	P15289	Arylsulfatase A (ASA) (EC 3.1.6.8) (Cerebroside-sulfatase) [Cleaved into: Arylsulfatase A component B; Arylsulfatase A component C]
− 0.99	0.24	0.01	P61421	V-type proton ATPase subunit d 1 (V-ATPase subunit d 1) (32 kDa accessory protein) (V-ATPase 40 kDa accessory protein) (V-ATPase AC39 subunit) (p39) (Vacuolar proton pump subunit d 1)
− 0.56	0.70	0.00	P16070	CD44 antigen (CDw44) (Epican) (Extracellular matrix receptor III) (ECMR-III) (GP90 lymphocyte homing/adhesion receptor) (HUTCH-I) (Heparan sulfate proteoglycan) (Hermes antigen) (Hyaluronate receptor) (Phagocytic glycoprotein 1) (PGP-1) (Phagocytic glycoprotein I) (PGP-I) (CD antigen CD44)
− 1.21	0.63	0.00	P08962	CD63 antigen (Granulophysin) (Lysosomal-associated membrane protein 3) (LAMP-3) (Melanoma-associated antigen ME491) (OMA81H) (Ocular melanoma-associated antigen) (Tetraspanin-30) (Tspan-30) (CD antigen CD63)
− 0.87	0.42	0.01	P12532	Creatine kinase U-type, mitochondrial (EC 2.7.3.2) (Acidic-type mitochondrial creatine kinase) (Mia-CK) (Ubiquitous mitochondrial creatine kinase) (U-MtCK)
− 0.95	0.81	0.00	P53634	Dipeptidyl peptidase 1 (EC 3.4.14.1) (Cathepsin C) (Cathepsin J) (Dipeptidyl peptidase I) (DPP-I) (DPPI) (Dipeptidyl transferase) [Cleaved into: Dipeptidyl peptidase 1 exclusion domain chain (Dipeptidyl peptidase I exclusion domain chain); Dipeptidyl peptidase 1 heavy chain (Dipeptidyl peptidase I heavy chain); Dipeptidyl peptidase 1 light chain (Dipeptidyl peptidase I light chain)]
− 1.00	0.50	0.01	P00167	Cytochrome b5 (Microsomal cytochrome b5 type A) (MCB5)
− 1.20	0.55	0.01	O75911	Short-chain dehydrogenase/reductase 3 (EC 1.1.1.300) (DD83.1) (Retinal short-chain dehydrogenase/reductase 1) (retSDR1) (Retinol dehydrogenase 17) (Short chain dehydrogenase/reductase family 16C member 1)
− 0.64	0.55	0.01	O60884	DnaJ homolog subfamily A member 2 (Cell cycle progression restoration gene 3 protein) (Dnj3) (Dj3) (HIRA-interacting protein 4) (Renal carcinoma antigen NY-REN-14)
− 1.04	0.74	0.00	Q9UK22	F-box only protein 2
− 1.57	0.84	0.00	P04066	Tissue alpha-L-fucosidase (EC 3.2.1.51) (Alpha-L-fucosidase I) (Alpha-L-fucoside fucohydrolase 1) (Alpha-L-fucosidase 1)
− 0.88	0.52	0.00	Q96C23	Aldose 1-epimerase (EC 5.1.3.3) (Galactose mutarotase)
− 0.84	0.67	0.00	Q9P2T1	GMP reductase 2 (GMPR 2) (EC 1.7.1.7) (Guanosine 5′-monophosphate oxidoreductase 2) (Guanosine monophosphate reductase 2)
− 0.82	0.86	0.00	P63096	Guanine nucleotide-binding protein G(i) subunit alpha-1 (Adenylate cyclase-inhibiting G alpha protein)
− 1.37	0.81	0.00	P15586	N-acetylglucosamine-6-sulfatase (EC 3.1.6.14) (Glucosamine-6-sulfatase) (G6S)
− 1.00	0.64	0.00	Q9NRV9	Heme-binding protein 1 (p22HBP)
− 2.02	0.21	0.00	P16401	Histone H1.5 (Histone H1a) (Histone H1b) (Histone H1 s-3)
− 1.70	0.35	0.01	P80365	Corticosteroid 11-beta-dehydrogenase isozyme 2 (EC 1.1.1.-) (11-beta-hydroxysteroid dehydrogenase type 2) (11-DH2) (11-beta-HSD2) (11-beta-hydroxysteroid dehydrogenase type II) (11-HSD type II) (11-beta-HSD type II) (NAD-dependent 11-beta-hydroxysteroid dehydrogenase) (11-beta-HSD) (Short chain dehydrogenase/reductase family 9C member 3)
− 3.15	0.32	0.00	P08729	Keratin, type II cytoskeletal 7 (Cytokeratin-7) (CK-7) (Keratin-7) (K7) (Sarcolectin) (Type-II keratin Kb7)
− 1.64	0.41	0.00	P11279	Lysosome-associated membrane glycoprotein 1 (LAMP-1) (Lysosome-associated membrane protein 1) (CD107 antigen-like family member A) (CD antigen CD107a)
− 1.56	0.61	0.00	P13473	Lysosome-associated membrane glycoprotein 2 (LAMP-2) (Lysosome-associated membrane protein 2) (CD107 antigen-like family member B) (LGP-96) (CD antigen CD107b)
− 1.43	1.04	0.00	O00462	Beta-mannosidase (EC 3.2.1.25) (Lysosomal beta A mannosidase) (Mannanase) (Mannase)
− 0.99	0.53	0.00	Q9H8H3	Methyltransferase-like protein 7A (EC 2.1.1.-) (Protein AAM-B)
− 1.45	0.34	0.01	Q92597	Protein NDRG1 (Differentiation-related gene 1 protein) (DRG-1) (N-myc downstream-regulated gene 1 protein) (Nickel-specific induction protein Cap43) (Reducing agents and tunicamycin-responsive protein) (RTP) (Rit42)
− 1.66	0.44	0.00	Q5TFE4	5′-nucleotidase domain-containing protein 1 (EC 3.1.3.-)
− 0.91	0.63	0.00	Q92882	Osteoclast-stimulating factor 1
− 1.21	1.36	0.00	Q15124	Phosphoglucomutase-like protein 5 (Aciculin) (Phosphoglucomutase-related protein) (PGM-RP)
− 0.29	0.49	0.01	A6NDG6	Glycerol-3-phosphate phosphatase (G3PP) (EC 3.1.3.21) (Aspartate-based ubiquitous Mg(2 +)-dependent phosphatase) (AUM) (EC 3.1.3.48) (Phosphoglycolate phosphatase) (PGP)
− 1.15	1.04	0.00	Q86T03	Type 1 phosphatidylinositol 4,5-bisphosphate 4-phosphatase (Type 1 PtdIns-4,5-P2 4-Ptase) (EC 3.1.3.78) (PtdIns-4,5-P2 4-Ptase I) (Transmembrane protein 55B)
− 0.95	0.44	0.01	Q8NHP8	Putative phospholipase B-like 2 (EC 3.1.1.-) (76 kDa protein) (p76) (LAMA-like protein 2) (Lamina ancestor homolog 2) (Phospholipase B domain-containing protein 2) [Cleaved into: Putative phospholipase B-like 2 32 kDa form; Putative phospholipase B-like 2 45 kDa form]
− 0.74	0.55	0.00	O15162	Phospholipid scramblase 1 (PL scramblase 1) (Ca(2 +)-dependent phospholipid scramblase 1) (Erythrocyte phospholipid scramblase) (MmTRA1b)
− 0.72	0.56	0.00	O15305	Phosphomannomutase 2 (PMM 2) (EC 5.4.2.8)
− 0.53	0.47	0.01	P35813	Protein phosphatase 1A (EC 3.1.3.16) (Protein phosphatase 2C isoform alpha) (PP2C-alpha) (Protein phosphatase IA)
− 1.20	0.63	0.00	P42785	Lysosomal Pro-X carboxypeptidase (EC 3.4.16.2) (Angiotensinase C) (Lysosomal carboxypeptidase C) (Proline carboxypeptidase) (Prolylcarboxypeptidase) (PRCP)
− 1.03	0.26	0.01	P11216	Glycogen phosphorylase, brain form (EC 2.4.1.1)
− 0.69	0.48	0.00	P61106	Ras-related protein Rab-14
− 0.68	0.78	0.00	Q9H0U4	Ras-related protein Rab-1B
− 0.58	0.51	0.00	Q9UL25	Ras-related protein Rab-21
− 0.80	0.44	0.01	P51149	Ras-related protein Rab-7a
− 1.64	0.18	0.00	P51151	Ras-related protein Rab-9A
− 0.69	0.31	0.01	P61224	Ras-related protein Rap-1b (GTP-binding protein smg p21B)
− 1.03	0.63	0.01	P61225	Ras-related protein Rap-2b
− 1.04	0.30	0.00	Q8TC12	Retinol dehydrogenase 11 (EC 1.1.1.300) (Androgen-regulated short-chain dehydrogenase/reductase 1) (HCV core-binding protein HCBP12) (Prostate short-chain dehydrogenase/reductase 1) (Retinal reductase 1) (RalR1) (Short chain dehydrogenase/reductase family 7C member 1)
− 0.69	0.61	0.01	Q14108	Lysosome membrane protein 2 (85 kDa lysosomal membrane sialoglycoprotein) (LGP85) (CD36 antigen-like 2) (Lysosome membrane protein II) (LIMP II) (Scavenger receptor class B member 2) (CD antigen CD36)
− 1.01	0.36	0.01	P01011	Alpha-1-antichymotrypsin (ACT) (Cell growth-inhibiting gene 24/25 protein) (Serpin A3) [Cleaved into: Alpha-1-antichymotrypsin His-Pro-less]
− 0.82	0.80	0.00	Q9HAT2	Sialate O-acetylesterase (EC 3.1.1.53) (H-Lse) (Sialic acid-specific 9-O-acetylesterase)
− 1.69	0.49	0.01	Q6IA17	Single Ig IL-1-related receptor (Single Ig IL-1R-related molecule) (Single immunoglobulin domain-containing IL1R-related protein) (Toll/interleukin-1 receptor 8) (TIR8)
− 0.78	0.77	0.00	Q00796	Sorbitol dehydrogenase (EC 1.1.1.14) (L-iditol 2-dehydrogenase)
− 1.60	0.50	0.00	Q13488	V-type proton ATPase 116 kDa subunit a isoform 3 (V-ATPase 116 kDa isoform a3) (Osteoclastic proton pump 116 kDa subunit) (OC-116 kDa) (OC116) (T-cell immune regulator 1) (T-cell immune response cDNA7 protein) (TIRC7) (Vacuolar proton translocating ATPase 116 kDa subunit a isoform 3)
− 0.29	1.17	0.01	Q9UGI8	Testin (TESS)
− 0.58	0.62	0.01	O76062	Delta(14)-sterol reductase (Delta-14-SR) (EC 1.3.1.70) (Another new gene 1 protein) (C-14 sterol reductase) (Putative sterol reductase SR-1) (Sterol C14-reductase) (Transmembrane 7 superfamily member 2)
− 1.92	0.05	0.01	Q9NUM4	Transmembrane protein 106B
− 0.71	0.38	0.00	Q12792	Twinfilin-1 (Protein A6) (Protein tyrosine kinase 9)
− 0.40	1.13	0.00	O15498	Synaptobrevin homolog YKT6 (EC 2.3.1.-)

In order to classify the differentially regulated proteins in RO or chRCC tissue, we performed a gene ontology (GO) enrichment analysis, with a focus on the “cellular compartment” annotation [27, 28]. We chose the TopGO algorithm to minimize GO term redundancy [29–31]. For RO tissue, this analysis points towards an enrichment of mitochondrial proteins whereas lysosomal proteins appear to be enriched in chRCC tissue (Fig. 2b**)**. High abundance of—potentially respiration-defective—mitochondria has been previously named as a distinguishing feature of renal oncocytomas [11, 32, 33]. Likewise lysosomal defects have been identified as a hallmark of ROs [11]. Likewise, an under-representation of endocytotic proteins in ROs has been reported [12] and the presence of vesicular proteins of the tetraspanin family has been proposed as a chRCC marker [8]. We conclude that our proteome profiling reflects hallmark features of ROs and chRCCs.

### Differential expression of LAMP1 in renal oncocytomas and chromophobe renal cell carcinoma

**Fig. 3:**
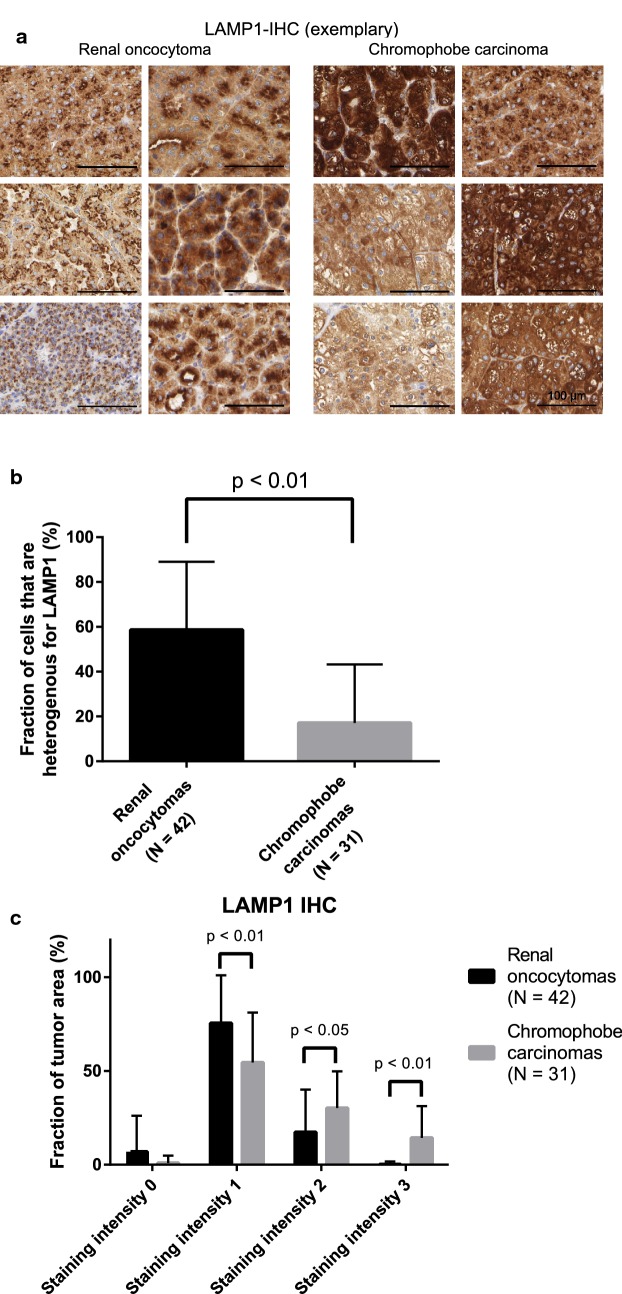
**a** Exemplary immunohistochemistry (IHC) of LAMP1 in RO and chRCC. **b** Fraction of RO or chRCC cells displaying a heterogenous staining pattern for LAMP1 (statistical significance determined using the two-way Student t-test; values are mean ± standard deviation). **b** Overview of LAMP1 IHC in the extended cohort using a semi-quantitative scoring system covering absence of intensity (score 0), weak detection (score 1), medium detection (score 2), and strong detection (score 3). Statistical significance determined using the two-way Student t-test; values are mean ± standard deviation; scale bar is 100 μm

Lysosomal defects have been reported for ROs [34] but lysosomal marker proteins have not yet been used to distinguish ROs and chRCCs by IHC. In our proteomic dataset, we noticed significant enrichment of lysosome-associated membrane proteins (LAMPs) 1– 3 in chRCC (Table 3). Of these, we chose LAMP1 for IHC analysis in an extended cohort comprising 42 RO cases and 31 chRCC cases. Although LAMP-2 and -3 were even stronger enriched in chRCCs, we opted for LAMP-1 since we had previously probed LAMP-1 as a prototypical marker of lysosomal biology [35, 36]. Exemplary stainings are shown in Fig. 3a. In the RO cases, we noticed elevated levels of tumor cells with a heterogenous distribution of LAMP1, comprising single or multiple LAMP-1 clusters with focal or apical localization. In contrast, LAMP1 within chRCC tumor cells was often present in a diffuse cytosolic manner (Fig. 3a, b). Similarly different staining patterns (diffuse in chRCC; apical/polar in RO) have been previously reported for the related protein LAMP-3 (referred to as CD63 in the study) [4, 37]. In addition to the different localization, we used a semi-quantitative scoring system to evaluate the LAMP1 presence, covering absence of signal (score 0), weak detection (score 1), medium detection (score 2), and strong detection (score 3). For cells displaying heterogenous LAMP1 staining, the predominant staining intensity was considered; this typically being the weaker signal. Our analysis highlighted that ROs are characterized by weaker LAMP1 presence in contrast to the elevated LAMP1 presence in chRCCs (Fig. 3c). The IHC analysis thus corroborates the proteomic result.

To assess a possible correlation of LAMP1 staining with clinical-pathological parameters of chRCC such as overall survival and T1–T4 staging, we are referring to data of the The Human Protein Atlas/Pathology Atlas [38, 39] since our cohort focuses on T1 and T2 stages. LAMP1 showed a tendency for shortened overall survival upon elevated expression but no correlation with tumor stage.

### Differential expression of ITGAV in renal oncocytomas and chromophobe renal cell carcinomas

**Fig. 4:**
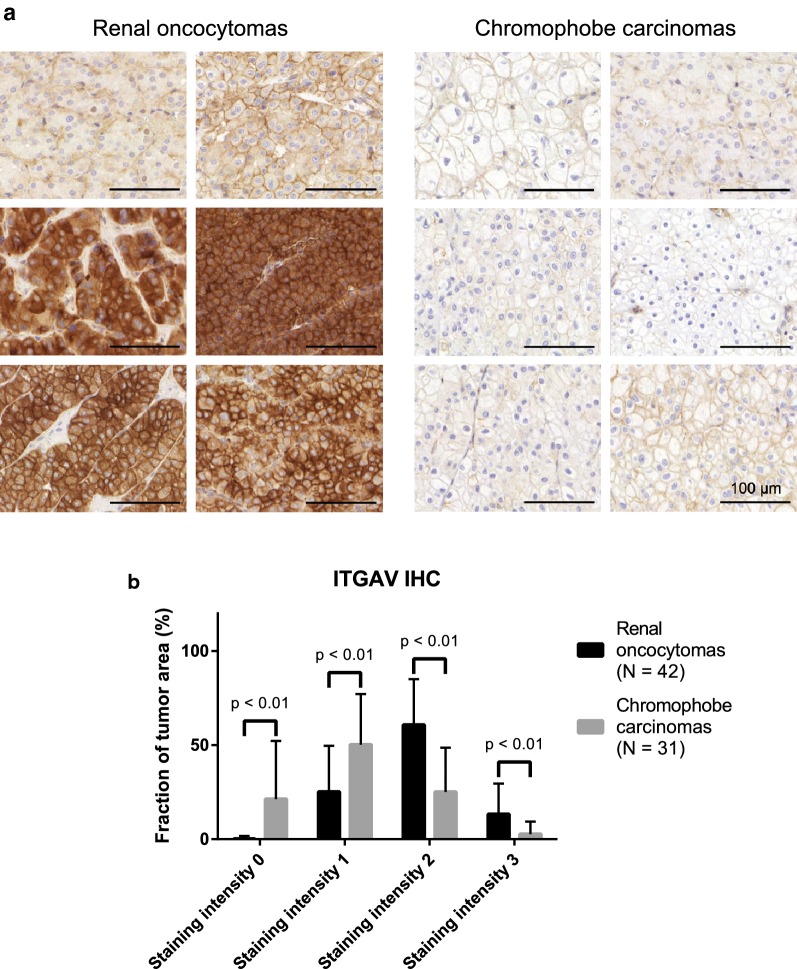
**a** Exemplary immunohistochemistry (IHC) of ITGAV in RO and chRCC. **b** Overview of ITGAV IHC in the extended cohort using a semi-quantitative scoring system covering absence of intensity (score 0), weak detection (score 1), medium detection (score 2), and strong detection (score 3). Statistical significance determined using the two-way Student t-test; values are mean ± standard deviation, scale bar is 100 μm

Integrin biology has been rarely reported as a differentiating feature that discriminates RO and chRCC. In our proteomic data, we noticed significant enrichment of ITGAV in the RO cases, which we further investigated by IHC in the extended IHC cohort (see above). In good agreement with the proteomic data, there was comparably strong presence of ITGAV in the RO cases while ITGAV presence in chRCC was sparse (Fig. 4a). This was further corroborated by a semi-quantitative analysis (see above for details) of different staining ITGAV staining intensities and their fraction of the tumor area under investigation (Fig. 4b).

Several transcriptome studies found comparable transcript levels of ITGAV, both when comparing RO and chRCC as well as when extending the comparison to other renal neoplasms [8, 12, 40, 41]. However, as previously outlined, there is increasing evidence that mRNA abundance and protein levels only display a limited correlation [13, 14], e.g. due to differential synthesis and degradation rates, thus emphasizing the importance of direct protein analysis by means of mass spectrometry or immuno-detection.

To assess a possible correlation of ITGAV staining with clinical-pathological parameters of chRCC such as overall survival and T1–T4 staging, we are referring to data of the The Human Protein Atlas/Pathology Atlas [38, 39] since our cohort focuses on T1 and T2 stages. ITGAV showed a tendency for shortened overall survival upon elevated expression but no correlation with tumor stage.

## Conclusion

We present one of the first proteomic profiling studies to differentiate renal oncocytomas and chromophobe renal cell carcinomas. We found distinguishable proteome profiles, which reflect previously annotated, discriminating features of ROs and chRCCs. Moreover, we identified novel protein candidates for which differential expression between ROs and chRCCs has not yet been described. Using an extended cohort of > 70 RO and chRCC cases, we corroborate strong presence of ITGAV in RO and of LAMP1 in chRCC. Methodologically, our work further validates the robustness of using FFPE material for retrospective quantitative proteomics as a first step for differential marker identification. Extension to further variants of renal cell neoplasms [32] is an intriguing outlook.

## Additional files

**Additional file 1: Table S1.** Labeling scheme setup.

**Additional file 2: Table S2.** Overview of the > 2400 proteins were identified (false-discovery rate < 1%) and quantified in at least four RO and four chRCC samples.

## Abbreviations

ccRCC: cell renal cell carcinoma
chRCC: chromophobe renal cell carcinoma
FFPE: formalin-fixed, paraffin-embedded
IHC: immunohistochemistry
ITGAV: integrin alpha-V
LAMP1: lysosomal-associated membrane protein 1
LC–MS/MS: liquid chromatography–tandem mass spectrometry
RO: renal oncocytomas
